# Effectiveness of the *Leptospira* Hardjo Control Programme and Detection of New Infections in Dairy Cattle in The Netherlands

**DOI:** 10.3390/ani13050831

**Published:** 2023-02-24

**Authors:** Katrien M. J. A. van den Brink, Marian Aalberts, Nannet D. Fabri, Inge M. G. A. Santman-Berends

**Affiliations:** 1 Department of Cattle Health, Royal GD, 7400 AA Deventer, The Netherlands; 2 Department of Research and Development, Royal GD, 7400 AA Deventer, The Netherlands

**Keywords:** leptospirosis, *Leptospira* serovar hardjo, control programme, purchase, risk factor, disease control, surveillance, dairy, serology, import

## Abstract

**Simple Summary:**

Leptospirosis in cattle is a zoonotic bacterial disease for which participation in a control programme is mandatory for dairy herds in the Netherlands. Nearly all dairy herds have an *L*. Hardjo-free status, and only sporadic cases of this disease occur on dairy farms. In this study, the effectiveness of the programme for early detection of new *L.* Hardjo infections between 2017 and 2021 were evaluated. During the analysed period, a suspected infection was detected 144 times in 120 dairy herds. Of these 144 introductions, only 26 lead to within-herd transmission of Leptospirosis. The main cause of the introduction of the disease was the purchase of cattle from herds without an *L.* Hardjo-free status. Additionally, there were no cases where the infection seemed to have spread locally. It was, therefore, concluded that the control programme was highly effective in the early detection and subsequent control of new infections.

**Abstract:**

Since 2005, a mandatory *L.* Hardjo control programme (LHCP) has been in place for Dutch dairy herds. Almost 100 percent of dairy farms participate and have an *L*. Hardjo-free status. In 2020 and 2021, the number of outbreaks seemed to increase as compared to the previous years. In this study, we evaluated the effectiveness of the national LHCP in the Netherlands during 2017–2021. Cases of new infections in herds with an *L.* Hardjo-free status in the LHCP were described, including the role of risk factors for the introduction. Both the percentage of dairy herds with an *L.* Hardjo-free status that purchased cattle from herds without a free status and the number of purchased cattle increased over the years. A between-herd cluster evaluation showed that between 2017 and 2021, a suspected infection was detected 144 times in 120 dairy herds. In 26 cases (26 herds, 0.2%) new infections were identified, including within-herd transmission. No infection clusters were identified, indicating that infections never led to local transmission between dairy herds. The introduction of cattle from non-free herds appeared to be the cause of all *L.* hardjo infections in herds participating in the LHCP. Therefore, the national LHCP seems to be highly effective in the control of infections in dairy herds.

## 1. Introduction

Leptospirosis in cattle is predominantly caused by *Leptospira interrogans* serovar Hardjo type prajitno and *Leptospira borgpetersenii* serovar Hardjo type bovis. Both serovars have zoonotic potential, mainly for farmers and farm workers [1]. To our knowledge, serovar Hardjo-type prajitno has not been reported in cattle in the Netherlands, but serovar Hardjo-type bovis has been described in both cattle and cattle farmers in the Netherlands [2,3,4,5,6,7].

Leptospira infections can be acquired via direct or indirect contact with the urine of infected individuals or with contaminated water. The pathogen enters the body through mucous membranes, skin cuts, or abrasions [8,9]. Possible clinical signs of leptospirosis in cattle include abortion, infertility, and a poor milk yield, but infections can also remain asymptomatic [10,11]. The clinical signs of human *L.* Hardjo infections include febrile illness, pulmonary haemorrhage, and renal failure [9]. *L*. Hardjo infections in cattle can be detected in body fluids, including serum, milk, and urine. Direct detection methods, such as culture and polymerase chain reaction (PCR), may fail to detect intermittent shedding of leptospires by chronically infected carriers. Antibody tests, including microscopic agglutination tests (MAT) and enzyme-linked immunosorbent assays (ELISA), are, therefore, more effective to support a clinical diagnosis in cattle and to determine disease prevalence in herds. MAT is the World Organization for Animal Health reference test for cattle, and is often required for export purposes, but serological responses can be missed in chronic cases. ELISA is a more sensitive method that shows persistent seroresponses, which can also be used to detect *L.* Hardjo antibodies in individual or bulk milk samples [12,13,14,15].

The main risk factors for the introduction of *L.* Hardjo into dairy herds are contact with the urine of newly introduced infected animals into a herd (for example, after purchase) and indirect local transmission by, for example, contaminated surface water [16,17,18].

In 1989, the herd seroprevalence of leptospirosis in dairy cattle in the Netherlands was 35% [19]. A voluntary control programme with the aim to eliminate this zoonotic disease from individual cattle herds has been in place in the Netherlands since 1994 [19,20]. This *L.* Hardjo-free control programme (LHCP) is based on testing for *L.* Hardjo antibodies in serum and bulk milk [21] and is carried out by Royal GD (Deventer, the Netherlands). An *L.* Hardjo-certified free status has been required by the Dutch Dairy Board for herds delivering milk to Dutch dairy plants since 2005. Currently (2022), 99.9% of all Dutch dairy herds and 33% of non-dairy herds participate in the LHCP. The vast majority of these herds are classified as being *L.* Hardjo-free [18]. Since *L.* Hardjo-free status became obligatory for dairy herds only sporadic introductions occurred [21], with the exception of the years 2020 and 2021, when an increased number of introductions of *L.* Hardjo were identified [22,23]. Leptospirosis is a notifiable disease in the Netherlands. Therefore, introductions of *L.* Hardjo are registered in the Dutch Cattle Health Surveillance system (CHSS) [24].

The aim of the study reported in this article was to evaluate the effectiveness of the national LHCP in the Netherlands in the context of early detection of new infections and prevention of transmission of *L.* Hardjo between herds between 2017 and 2021. Cases of new infections of *L.* Hardjo in dairy herds with an *L.* Hardjo-free status were described, including the role of known risk factors for introduction, such as livestock purchases and local between-herd transmission.

## 2. Materials and Methods

### 2.1. L. Hardjo-Free Control Programme

Although both dairy and non-dairy herds participated in the LHCP, we focused on the population of approximately 15,000 participating Dutch dairy herds over the period from 2017 to 2021. In 2021 there were 1.57 million dairy cows registered in the Netherlands, with an average herd size of 110 cows (>2 years old). This included almost 100% of the Dutch dairy herds and only excluded a very small number of herds that did not sell their milk to dairy plants that are members of the Dutch Dairy Board. The total number of dairy herds in the Netherlands declined during the study period, resulting in a decreasing number of participants. The LHCP was described in detail by Santman-Berends et al. [21]. In short, herds are assigned the *L.* Hardjo-free status after an initial assessment with all negative test results consisting of individual *L.* Hardjo antibody testing. Surveillance of the subsequent *L.* Hardjo-free dairy herds is based on *L.* Hardjo antibody testing of (1) bulk milk samples three times a year, (2) post-movement sera from cattle introduced from herds without *L.* Hardjo-free status, (3), and clinically suspected animals, including aborted cattle. Mandatory serum testing of introduced animals from non-free herds is assigned directly after the introduction of animals into the receiving herd and has to be completed within eight weeks. *L.* Hardjo antibody tests in bulk milk and sera are performed using the PrioCHECK *L.* Hardjo Ab Plate Kit (Thermo Fisher Scientific, Applied Biosystems, Lelystad, the Netherlands) [12]. For both the serum and bulk milk ELISA, sensitivity is >95%, and specificity is >99%.

**Figure 1:**
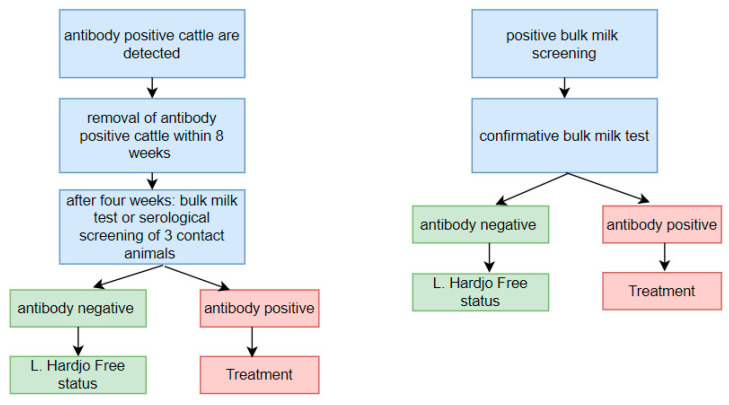
Control actions following detection of antibody positive cattle or bulk milk screening in the *Leptospira* Hardjo Control Programme.

In the event of a positive or inconclusive antibody test result of the introduced cattle or clinically suspect cattle, the *L.* Hardjo-free status of the receiving herd is suspended, and confirmatory testing is mandatory (Figure 1). Seropositive animals have to be removed from the herd, followed by a bulk milk test four weeks later or by serological testing of three contact animals, to attain certified-free status. In case of a positive or inconclusive outcome of this subsequent testing, individual testing of all animals in the herd is mandatory to investigate possible transmission of *L.* Hardjo within the receiving herd. In the event of a positive bulk milk screening test, the *L.* Hardjo-free status is suspended, and a second bulk milk sample is taken for confirmation of transmission. If the transmission is confirmed, the herd status is changed to ‘infected’, and all animals in the herd are treated on a single day with a single intramuscular dose of dihydrostreptomycin [3]. All participating herds in the LHCP that purchased cattle from an infected herd are notified to perform post-movement serum testing of those cattle. After treatment, the herd status changes to ‘controlled’. To survey for transmission of *L.* Hardjo in herds with a ‘controlled’ status, a seronegative sentinel group of five animals of ≥2 years old are tested serologically every six months. A herd can retrieve the *L.* Hardjo-free status after a new assessment of individual serum tests of all animals >1 month of age.

### 2.2. Definitions

The following definitions were used:

- **Purchase:** Introduction of an animal into a herd from a Dutch herd, whether or not a financial transaction occurred;
- **Import:** Introduction of an animal into a herd from a herd located outside the Netherlands;
- **High risk animals:** Animals introduced from herds not participating in the LHCP, or from herds participating in the LHCP without an *L.* Hardjo-free status;
- **Suspended herd status:** Temporary loss of a certified-free status requiring action to regain the herds’ free status. There are multiple reasons for suspending the certified free status, such as a delay in submitting required samples, introduction of new cattle, or a positive test result;
- **Suspected herd:** A herd that is suspected to have an *L.* Hardjo infection, indicated by an antibody positive serum or bulk milk sample, and mandatory confirmatory testing to detect spread has not been completed yet;
- **Infected herd:** A herd in which spread of *L*. Hardjo was proven by two positive *L.* Hardjo antibody tests in consecutive bulk milk samples (within a period of eight weeks), or an *L.* Hardjo antibody positive serum test result followed by either a positive bulk milk test result, or three serum samples taken four weeks after removal of the positive animal (Figure 1). This is also referred to as a new infection in this manuscript;
- **Neighbouring herds:** Infected and/or suspected herds that were located within a 5 km radius of each other;
- **Cluster:** A group of neighbouring herds with an overlapping maximum potential infectious period (see Section 2.4 for more detail).

### 2.3. Data Collection and Study Period

Herd data and *L.* Hardjo status were obtained from an automated certification administration system at GD. This system receives near real-time (daily) cattle movement information from the national identification and registration database (Netherlands Enterprise agency, Assen, the Netherlands). The movement data, combined with LHCP status data, enabled identification of cattle transfers from non-free herds during the period 2017–2021.

*L*. Hardjo status of herds could change during the study period. The total number of participants of the programme, and status data of participants, were therefore described for the last day of each of the evaluated years. Risk factors, including purchase, import, and local transmission, were evaluated by using data on numbers of introduced cattle, the *L.* Hardjo status of the herds of origin, and dates of introduction. Herds of origin not participating in the LHCP could be located either outside or within the Netherlands. If the *L*. Hardjo status from the herd of origin was unknown, a possible positive *L.* Hardjo herd status was assumed, and the introduced cattle were characterized as high-risk animals. The proportion of certified *L.* Hardjo-free herds that introduced high risk animals, and the number of high-risk animals introduced per herd per year, are descriptively presented. A chi-square test was used to evaluate whether or not the average number of high-risk cattle introduced into a herd varied between years.

### 2.4. Cluster Evaluation

**Figure 2:**
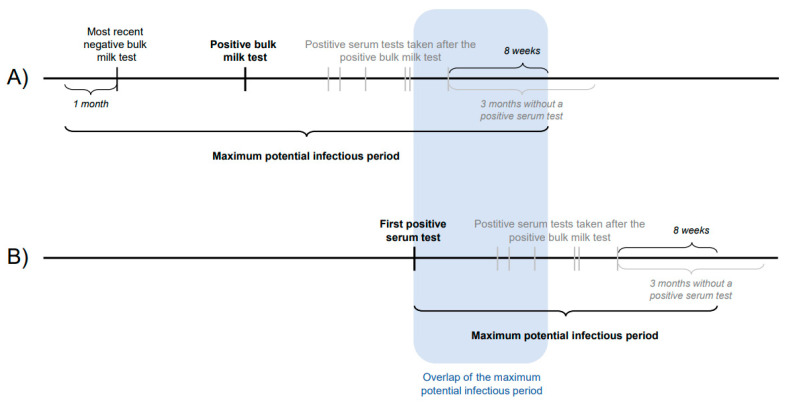
Graphical depiction of the definition of the maximum potential infectious period of a herd, if it became suspected due to (**A**) a positive bulk milk sample or (**B**) a positive serum sample.

Cluster evaluation was used to investigate transmission between neighbouring herds. Herds with a suspected and infected status were included. Herds within a 5 km radius of each other were classified as neighbouring herds. For each neighbouring herd the duration of the maximum potential infectious period was calculated. A herd was classified as suspected if either a milk sample or a serum sample tested positive. For herds with a positive bulk milk sample, the maximum potential infectious period was defined as one month before the last negative bulk milk sample until eight weeks after the last positive sample that was taken in response to the positive bulk milk sample (Figure 2). The limit of eight weeks was chosen because, in the LHCP, positive animals have to be removed from the herd within eight weeks after confirmation of an *L.* Hardjo infection. For herds where only positive serum samples were found, it was not known when the positive animals would have become infectious. Therefore, based on a worst- and best-case scenario, two maximum potential infectious periods were defined. In the best case, the start of the maximum potential infectious period was assumed to start on the day the first positive sample was taken. In the worst case, the start was assumed to be eight weeks earlier. This time frame was chosen because, in the LHCP, a high-risk animal must be tested within eight weeks after introduction. In both cases, the maximum potential infectious period ended eight weeks after the last positive serum sample was taken (Figure 2). Overlap in time and space of their maximum potential infectious periods were described for all neighbouring herds. If there was an overlap, both herds were grouped into the same cluster.

The time of purchase or importation of cattle, particularly whether or not it occurred within the preceding year, was determined for each herd in the identified clusters. This indicated if the most probable route of introduction of infection was through purchase, import, or otherwise (for example, local transmission). It was also assessed whether or not the identified clusters remained if the 5 km radius was reduced to 3 km or 1 km. All analyses in the cluster evaluation were performed in R-studio version 4.1.3 [25] with the aid of the libraries lubridate, data.table, and tidyverse for analytical purposes [26,27,28].

## 3. Results

### 3.1. Participation in the LHCP

**Figure 3:**
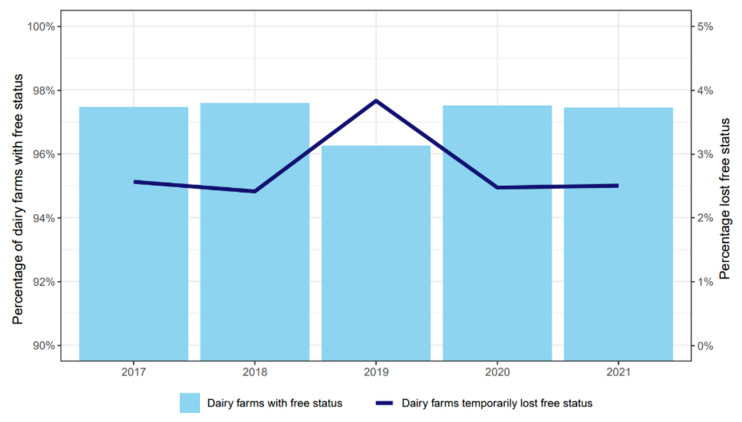
Percentages of dairy herds participating in the *Leptospira* Hardjo Control Programme with the free status (light blue columns) and the percentage that temporarily lost the status from 2017 to 2021 (dark blue line).

On average, 97% of the Dutch dairy herds participating in the LHCP had an *L.* Hardjo-free status between 2017 and 2021 (Figure 3). The remaining 3% of the herds had a temporary suspension of their free status due to the need to provide evidence of freedom, either because of the purchase of animals or a delay to comply with the LHCP surveillance scheme. In sporadic cases, herds temporarily lost their free status due to a new infection of *L*. Hardjo.

**Figure 4:**
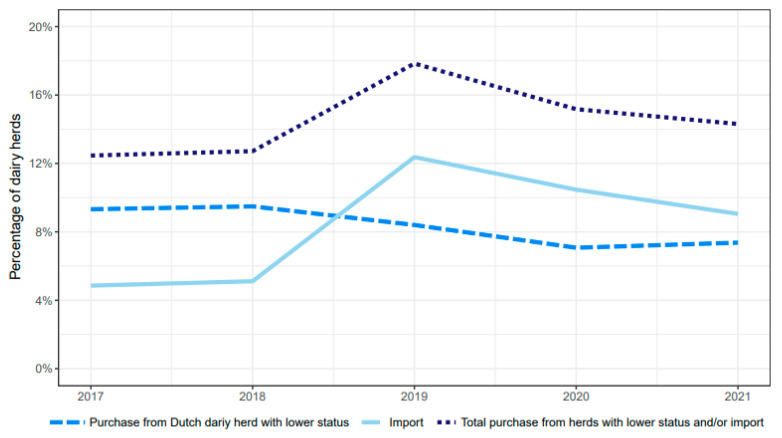
Percentages of dairy herds with a *L.* Hardjo-free status that purchased cattle from either herd with a lower *L.* Hardjo-free status and/or from herds abroad per year from 2017 to 2021.

Both the percentages of dairy herds with an *L.* Hardjo-free status that imported cattle, and the total number of imported or purchased cattle, increased over the investigated years, with a peak in 2019 (Figure 4). The proportion of purchases or importations of high-risk cattle, in relation to the total purchases and importations, did not change during the study period. Of all herds that purchased or imported high-risk cattle, 53% performed this only once, 21% twice, 25% 3–10 times, and 1,5% more than 10 times per year.

**Figure 5:**
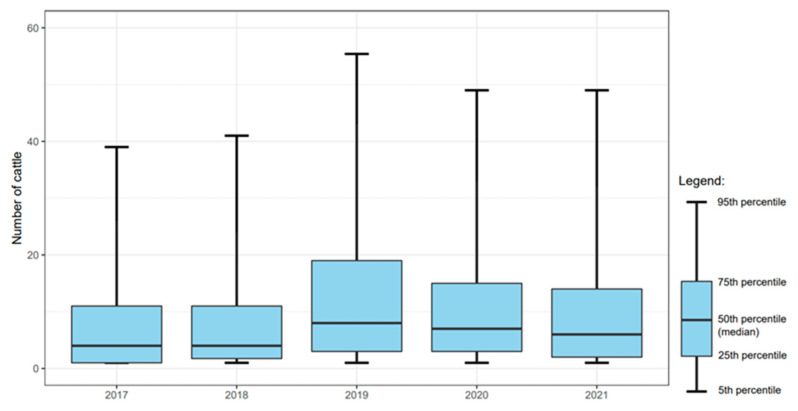
Box-and-Whisker plot of the numbers of introduced cattle per purchasing herd per year in L. Hardjo-free dairy herds originating from herds with a lower *L.* Hardjo status (including import).

The number of introduced high-risk cattle per event was significantly (χ^2^: *p* < 0.05) higher in 2019 (15.9), 2020 (13.9), and 2021 (12.9) compared to 2017 and 2018 (10.5 and 11.0, respectively) (Figure 5).

### 3.2. Cluster Evaluation

During the study period, 120 dairy herds, out of approximately 15,000 participating dairy herds, were classified as a suspected or infected herd at, in total, 144 occasions (Table 1). Of all the 120 herds with either a suspected or infected status, 21 herds were identified as herds where the infection overlapped in time and space. This was the case for both the best- and worst-case scenarios for herds with only positive serum samples. Therefore, the results are only presented once. Nine clusters of neighbouring herds were identified (<5 km) (Table 1). If this distance was reduced to 3 km, only four clusters remained, and with a distance of 1 kilometre, no clusters remained.

Positive serum samples were taken from animals that were imported recently at 12 of the 21 clustering herds. In 7 other of these 21 herds, positive samples were taken from animals that were purchased from herds in the Netherlands that were not, or not yet, assigned *L.* Hardjo-free status. For the remaining two herds, no link could be found with the recent introduction of cattle. These two herds belonged to two different clusters.

### 3.3. New Infections with L. Hardjo

Of the 120 herds that were suspected of infection or infected, 26 were classified as infected in the LHCP, given that within-herd transmission was observed. These 26 herds were not neighbouring herds and did not have *L.* hardjo infections that overlapped in time and space. Most of these were detected in 2020 and 2021. These 26 herds were not part of any cluster between 2017 and 2021 (Table 2).

#### 3.3.1. Introduction of Animals from Herds Not Participating in the LHCP

In twelve of the 26 cases, the infection was likely to be introduced into the herd by the introduction of imported animals. In seven of these cases, antibodies were detected by mandatory serological screening of recently introduced animals. In five other herds, the imported animals were all antibody-negative during mandatory screening, but the following bulk milk samples were antibody positive.

Three of the infected herds had recently introduced cattle that were purchased from Dutch (non-dairy) herds that did not participate in the LHCP. The sera that were tested for mandatory screening tested *L.* Hardjo antibody-positive, and the spread of the infection occurred after the introduction of the antibody-positive animals.

#### 3.3.2. Introduction of Animals from Dutch Herds Participating in the LHCP

In nine cases, the introduction of leptospirosis occurred after the purchase of cattle from *L.* Hardjo-infected farms. Eight of these nine cases followed purchases from one single herd (Herd A). In this infected herd, imported cattle had been introduced previously and were screened four days after their introduction into the herd, resulting in negative tests. Therefore, the free status of Herd A was initially retained. However, in the next bulk tank screening of this herd *L*. Hardjo antibodies were detected. In the period between the last negative bulk milk screening and the result of this positive bulk milk test, the farm sold cows with *L.* Hardjo-free status. A herd receives the “infected” status only when a positive bulk milk sample is confirmed, and all purchasing herds are notified to serum test purchased cattle that might be infected with *L.* Hardjo. However, before receiving the result of the positive bulk milk test, the farmer stopped milking and sold all the cows, which were then moved to different herds. No confirmation of the first positive bulk milk sample could be performed. Without a confirmative test result, the herds’ *L.* Hardjo status did not change from suspended to “infected”, according to the LHCP regulations. The farmers that purchased cattle from Herd A during the *L.* Hardjo-free status were, therefore, not initially notified to screen the purchased animals for antibodies against *L.* Hardjo. However, after the detection of leptospirosis in one of these herds, all participants in the LHCP that purchased cattle from Herd A were advised to perform serological screening, which resulted in a total of eight infected farms.

In the ninth case, a cow was imported and tested seropositive in the subsequent mandatory screening. This cow was then sold before treatment of the herd and introduced into another dairy herd. In both herds in which the animal was introduced, the infection was detected in the mandatory serum test and confirmed by a bulk milk test result.

#### 3.3.3. Cases Not Related to Introduction of Cattle

In two herds with new infections, no high-risk cattle were recently introduced. In the first case, a group of 30 heifers broke out to a neighbouring non-dairy herd that did not participate in the LHCP. In the second case, the herd was located at the same address as a non-dairy herd that did not participate in the LHCP. In both cases, leptospirosis may therefore have been introduced via local transmission. This hypothesis could unfortunately not be substantiated as the non-dairy herds were not screened for the presence of *L.* Hardjo.

## 4. Discussion

The findings of this study showed that, during the five-year study period, a suspected introduction was detected 144 times in 120 dairy herds that participated in the LHCP. Due to the surveillance scheme in place in the LHCP and quick actions taken by farmers; furthermore, the within-herd transmission was prevented in the majority of these herds. In our study, imported vaccinated animals could have been marked as animals with suspected infection in the study period, which may have led to an overestimation of the number of suspected infections. Within-herd transmission occurred in 26 herds participating in the LHCP in the Netherlands in the five-year study period, which was only 0.2% of the total number of participants (approximately 15,000).

In almost all cases, leptospirosis was found to be introduced into the herds via the introduction of high-risk cattle originating from herds that did not participate in the LHCP (import and purchase) or without an *L.* Hardjo-free status. The introduction of cattle from herds that were not certified-free thus appeared to be the most important risk factor for the introduction of *L.* hardjo in herds participating in the LHCP in the Netherlands. In the years 2019–2021, more herds either imported or purchased cattle from herds without an *L.* Hardjo-free status compared to 2017–2018, which was most likely associated with new Dutch legislation that resulted in less youngstock held per herd. The increased occurrence of importations and purchases led to a slight increase in the number of new infections.

In the cluster evaluation, a standardized definition for the maximum potential infectious period was used. By using this standardized definition, a worst-case situation was assumed as measures taken by farmers to remove infected animals were not taken into account. Nevertheless, the fast removal of positive animals reduces the maximum potential infectious period. In the cluster evaluation for one cluster was seen that the introduction of leptospirosis could not be directly linked to importations or purchases from herds without an *L.* Hardjo-free status. Upon closer examination, it appeared that in the first herd of this cluster, measures were taken to remove infected animals, thus reducing the maximum potential infectious period. By reducing this period, the two suspected herds did not have an overlapping maximum potential period anymore. For all other herds in the clusters, the introduction of leptospirosis could be directly linked to importations or purchases from herds without an *L*. Hardjo-free status. Therefore, local transmission of leptospirosis (with serotype Hardjo) between dairy herds participating in the LHCP was not observed and appeared unlikely. This suggests that the role of neighbouring farms was negligible in the transmission of *L*. Hardjo between Dutch dairy herds participating in the LHCP between 2017 and 2021.

The cluster evaluation only included dairy herds participating in the LHCP because the *L.* Hardjo status in non-participating herds was unknown. The non-dairy herds, for which participation in the LHCP was not required, often have an unknown *L.* Hardjo status and, therefore, could be a potential source of infection for local transmission. Nevertheless, the true *L.* Hardjo herd-level prevalence in the Dutch non-dairy sector is probably low. In the most recent national prevalence survey in 2013, only 0.8% of the non-dairy herds tested positive for *L*. Hardjo [22]. The risk for transmission from the non-dairy to the dairy sector is also assumed to be limited due to regulations in the LHCP.

Given the results of this study, we conclude that the LHCP is effective in the control of *L.* Hardjo in the Netherlands. Nevertheless, participation in the LHCP does not completely prevent new cases of leptospirosis. Therefore, some additional measures may be useful to minimize the risk of the introduction and spread of infection. For example, the interval and timing of testing could be optimized. Cattle infected and introduced from non-free herds may already transmit the pathogen before seroconversion and/or before the mandatory serum testing is performed [16], for example, when the animals contract the infection during the last month before the transfer or during transport. Moreover, it cannot be excluded that cattle may fail to respond serologically. In our study, we observed some cases in which the initial post-movement serological tests were negative after the introduction of high-risk animals. Nevertheless, at the subsequent routine surveillance sampling, which is part of the LHCP, the herd tested positive. As no other possible routes of infection were identified on these farms, the introduction of high-risk cattle seemed to be a plausible source of infection. In these cases, suspected infection was detected by regular bulk milk screening. Thus, if sera are taken <30 days after introduction, this may be too early to detect seroconversion [12]. In these specific cases, infections could have been detected earlier if the animals would have been (re)-tested >30 days after introduction into the herd. It is advised (but not mandatory) in the LHCP to serologically screen cattle before introduction into a herd (pre-movement testing), in addition to the mandatory screening after introduction [29]. It is, however, unknown how many herds perform pre-movement testing and how effective this would be. To further reduce the risk of transmitting the pathogen in the LHCP, animals might be quarantined until a negative test result of a second screening moment at >30 days after introduction into the herd. However, since testing twice and including additional quarantine measures is labour-intensive and costly, making these measures mandatory could reduce farmers’ compliance with the programme rules. Therefore, it was decided that these measures are not mandatory, but are advised in the LHCP [29]. If quarantine measures, in combination with testing at suggested times, would have been applied, the majority of the new infections found in 2017–2021 may have been prevented. However, these measures would probably not have been cost-efficient given the low prevalence and incidence of *L.* Hardjo in the Netherlands, resulting in only low economic losses.

It was the first time since 2005 that a case such as Herd A, where multiple farms became infected by one single farm within the LHCP, was observed. An additional measure to the LHCP regulations could be to notify purchasing herds that previously bought cattle from an *L.* Hardjo-free herd where a single positive bulk milk test cannot be confirmed by a second bulk milk test. The specific herd that was mentioned in this paper, Herd A, stopped milking, and when the first bulk milk sample was found, this result could not be substantiated with a second sample given that the herd was no longer producing milk, and thus purchasing farms were not notified. If in such cases, a single positive test result of regular bulk milk sampling would have been followed up by notifying the purchasing herds, the infections in the other eight herds may have been detected earlier. The purchasing herds could have been notified at an earlier stage to test the serum of the purchased animals. This could have prevented the eight infections related to the purchase from Herd A. This case represents a calamity within the LHCP, which could be prevented by implementing the improvements listed above. However, this case is currently considered to be a very rare event where there was an unlikely succession of coincidences.

Leptospirosis has a broad geographical distribution and, in some areas, for example, in tropical countries, it is an endemic disease, causing high mortality and morbidity [30,31]. In this manuscript, we focused on *L.* Hardjo in cattle in the Netherlands, but risk factors for leptospirosis may be different in other countries, and therefore, control of leptospirosis may require a different approach. For example, *Leptospira* serovars that circulate may differ in different parts of the world [32]. Survival of *Leptospira* in the environment may vary due to climate factors, surface water and soil composition, and different (vector) animal contact structures may exist [30]. Although leptospirosis is a difficult disease to control in the tropics, components of the LHCP could be implemented to control and possibly eradicate the disease on a herd level.

Vaccination is applied to control leptospirosis in cattle in several other countries [11,33]. However, vaccines for cattle against *L.* Hardjo are not registered in the Netherlands, and it would not be a cost-effective intervention in a situation where almost all dairy farms are certified free, and the incidence is at a very low level. High costs for vaccination, added to the costs involved with testing for participation in the LHCP, could result in a major loss of support for the LHCP by farmers. Additionally, vaccination would interfere with the ELISA tests used in the LHCP. This also argues against vaccination as an option in the Netherlands, although it has been shown that vaccination may be an effective measure in the control of Leptospirosis in other countries.

## 5. Conclusions

In this study, the effectiveness of the national LHCP in the Netherlands between 2017 and 2021 has been described with an emphasis on the early detection of new infections. Despite high numbers of imported and purchased cattle, the number of new infections remained low, with an incidence of 0.2% in the herds participating in the LHCP over a five-year period. Due to the early detection of new infections, the role of local between-herd transmission appeared minimal, as indicated by the fact that no clusters of infected herds in time and space were identified. It is therefore concluded that, although some calamities may occur sporadically, the national LHCP in the Netherlands is highly effective in the control of *L.* Hardjo infections.

## Figures and Tables

**Table 1:** Results of cluster evaluation of dairy herds participating in the *L.* Hardjo control programme. Clusters were classified according to the year in which the first positive sample occurred in the cluster.

Year	# Herds in LCHP	# Herds with ≥1 positive Test *	Cluster < 5 km		Cluster < 3 km		Cluster < 1 km	
			# Clusters	# Herds per Cluster	# Clusters	# Herds per Cluster	# Clusters	# Herds per Cluster
2017	16.355	10	1	2	0	-	0	-
2018	15.792	15	0	-	0	-	0	-
2019	15.295	47	4	4 3 2 2	3	4 2 2	0	-
2020	14.919	31	3	2 2 2	0	-	0	-
2021	14.450	26	1	2	1	2	0	-

* Herds with multiple positive tests per year were classified once in that year. Herds with positive test in multiple years were included in multiple years. Therefore, the sum of all herds with ≥1 positive test during the study period (129) is higher than the total number of unique herds with ≥1 positive test (120).

**Table 2:** Number of new infections of L. Hardjo from 2017 to 2021 in the Netherlands classified by most plausible introduction route.

Most Plausible Introduction Route	2017	2018	2019	2020	2021	2017–2021
Cattle purchased from herds not participating in LHCP *						
Import	0	1	2	6	3	12
Purchase	0	0	0	1	2	3
Cattle purchased from herds participating in the LHCP (regardless of status) *						
Purchase	0	0	1	6	2	9
Not related to introduction of cattle	0	1	0	1	0	2
Total of new infections	0	2	3	14	7	26

* In most cases, the introduction of cattle occurred in the same year as the detection of infection, for infections related to introduction of cattle.

## Data Availability

The herds or groups of herds included in this study may be identifiable from their unique combination of explanatory variables. Therefore, the datasets analysed in this study cannot, and will not, be made available to readers.
